# UV radiation recruits CD4^+^GATA3^+^ and CD8^+^GATA3^+^ T cells while altering the lipid microenvironment following inflammatory resolution in human skin *in vivo*

**DOI:** 10.1002/cti2.1104

**Published:** 2020-04-02

**Authors:** Nathan J Hawkshaw, Suzanne M Pilkington, Sharon A Murphy, Norah Al‐Gazaq, Mark D Farrar, Rachel EB Watson, Anna Nicolaou, Lesley E Rhodes

**Affiliations:** ^1^ Centre for Dermatology Research Division of Musculoskeletal and Dermatological Sciences Faculty of Biology, Medicine and Health School of Biological Sciences Manchester Academic Health Science Centre The University of Manchester and Salford Royal NHS Foundation Trust Manchester UK; ^2^ Laboratory for Lipidomics and Lipid Biology Division of Pharmacy and Optometry Faculty of Biology Medicine and Health School of Health Sciences The University of Manchester Manchester UK

**Keywords:** inflammation, immunosuppression, lipidomics, resolution, T cells, ultraviolet radiation

## Abstract

**Objectives:**

Solar ultraviolet radiation (UVR) has major adverse effects on human health. While the mechanisms responsible for induction of UVR‐induced inflammation are well‐documented, the mediation of its resolution and longer‐term adaptive homeostasis is unknown. Therefore, we examined the skin immune and lipid profile over time following UVR inflammation.

**Methods:**

To investigate the self‐resolving events of UVR inflammation *in vivo*, human skin was exposed to a single pro‐inflammatory dose of UVR. Skin biopsies and suction blister fluid were taken at intervals up to 2 weeks post‐UVR. The immune infiltrate was quantified by immunohistochemistry, and lipid mediators were profiled by liquid chromatography/mass spectrometry.

**Results:**

We identified that cellular resolution events including switching of macrophage phenotype apply to human sunburn. However, UVR‐induced inflammation in humans involves a post‐resolution phase that differs from other experimental models. We demonstrate that 2 weeks after the initiating UVR stimulus, there is considerable immune activity with CD8^+^GATA3^+^ T cells maintained in human skin. Our results challenge the dogma of CD4^+^FOXP3^+^ T cells being the main effector CD4^+^ T‐cell population following UVR, with CD4^+^GATA3^+^ T cells the dominant phenotype. Furthermore, lipid mediators are elevated 14 days post‐UVR, demonstrating the skin lipid microenvironment does not revert to the tissue setting occurring prior to UVR exposure.

**Conclusion:**

We have identified for the first time that CD4^+^GATA3^+^ and CD8^+^GATA3^+^ T‐cell subpopulations are recruited to UVR‐inflamed human skin, demonstrating discrepancies between the adaptive UVR response in mice and humans. Future strategies to abrogate UVR effects may target these T‐cell subpopulations and also the persistent alteration of the lipid microenvironment post‐UVR.

## Graphical abstract

Hawkshaw *et al.* demonstrate that, in the post‐resolution phase following acute UVR inflammation in human skin, CD8^+^GATA3^+^ cells are recruited and associated with an altered bioactive lipid profile. In contrast to murine models, CD4^+^GATA3^+^ T cells and not CD4^+^FOXP3^+^ cells predominate.

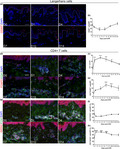

## Introduction

Solar ultraviolet radiation (UVR) has adverse effects on human health, which include induction of both inflammation (sunburn, photodermatoses) and immunosuppression of the skin (enhancing susceptibility to infection, reducing vaccination efficacy and is the major environmental risk factor for skin cancer).^1^ The increasing incidence of skin cancers leads to more medical consultations and treatment, with costs projected into the billions (USD) in the United States alone.^2^ Conversely, UVR‐induced immunosuppression in humans^3^ has beneficial effects in certain skin conditions and may benefit some systemic disorders.^4^ Therefore, it is clinically important to decipher how UVR modulates the skin’s immune and microenvironment, both to identify treatment targets and to guide public health strategies on sunlight exposure.^5^

Ultraviolet radiation induces self‐resolving inflammation (i.e. sunburn), characterised by erythema, pain and oedema ~4–6 h following prolonged exposure.^6^ While the cells and mediators responsible for the induction and peak of acute UVR‐induced inflammation in human skin have been extensively investigated,^7^ the longer‐term cellular and molecular events contributing to resolution and beyond are unknown.

Recent experimental investigations have identified that post‐inflammation, tissues do not revert to homeostatic conditions. Instead, ‘adaptive homeostasis’ occurs, where although the initiating inflammatory signal has subsided, a beneficial state of local immunosuppression remains.^8^ This post‐resolution phase is characterised by either a secondary infiltrate^9^, ^10^ or retention of immune cells following inflammatory resolution, and maintains an immunosuppressive environment.^8^ It is thought that regulatory T cells (Tregs) are central for cutaneous immunosuppression following UVR exposure in mice.^11^, ^12^ In addition, recent epidemiological evidence shows that the Treg subpopulation, CD45RA^−^/CD27^−^, in the peripheral blood of humans, positively correlates with a proxy of UVR exposure.^13^ However, there is no *in situ* evidence that UVR leads to the accumulation of Tregs in human skin.^13^ Moreover, the potential role of efferocytosis, a vital process for initiating inflammatory resolution,^14^ remains unexplored in the human cutaneous UVR response.

Pro‐inflammatory and pro‐resolving cellular events are not passive and require signals to coordinate their activity.^10^, ^14^, ^15^ Evidence from murine and human models supports that lipid mediators are required molecular signals at the onset and peak of inflammation, and may also direct resolution.^14^, ^15^, ^16^ Following UVR exposure in humans, synthesis of bioactive lipids occurs for 72 h.^17^ The activity of these lipid mediators in modulating UVR‐induced inflammation is evident as cyclooxygenase inhibitors reduce erythema.^18^ However, it is unknown whether a post‐resolution phase^8^ occurs following UVR and whether these lipid mediators participate. Therefore, the primary objective of this study was to examine the longer‐term cutaneous immune infiltrate and lipid profile of the sunburn response in healthy humans *in vivo*. This will reveal whether insights into UVR inflammation resolution gained from murine studies translate to the human system.

## Results and discussion

### Classical inflammatory cellular resolution occurs 4 days post‐UVR

**Figure 1:**
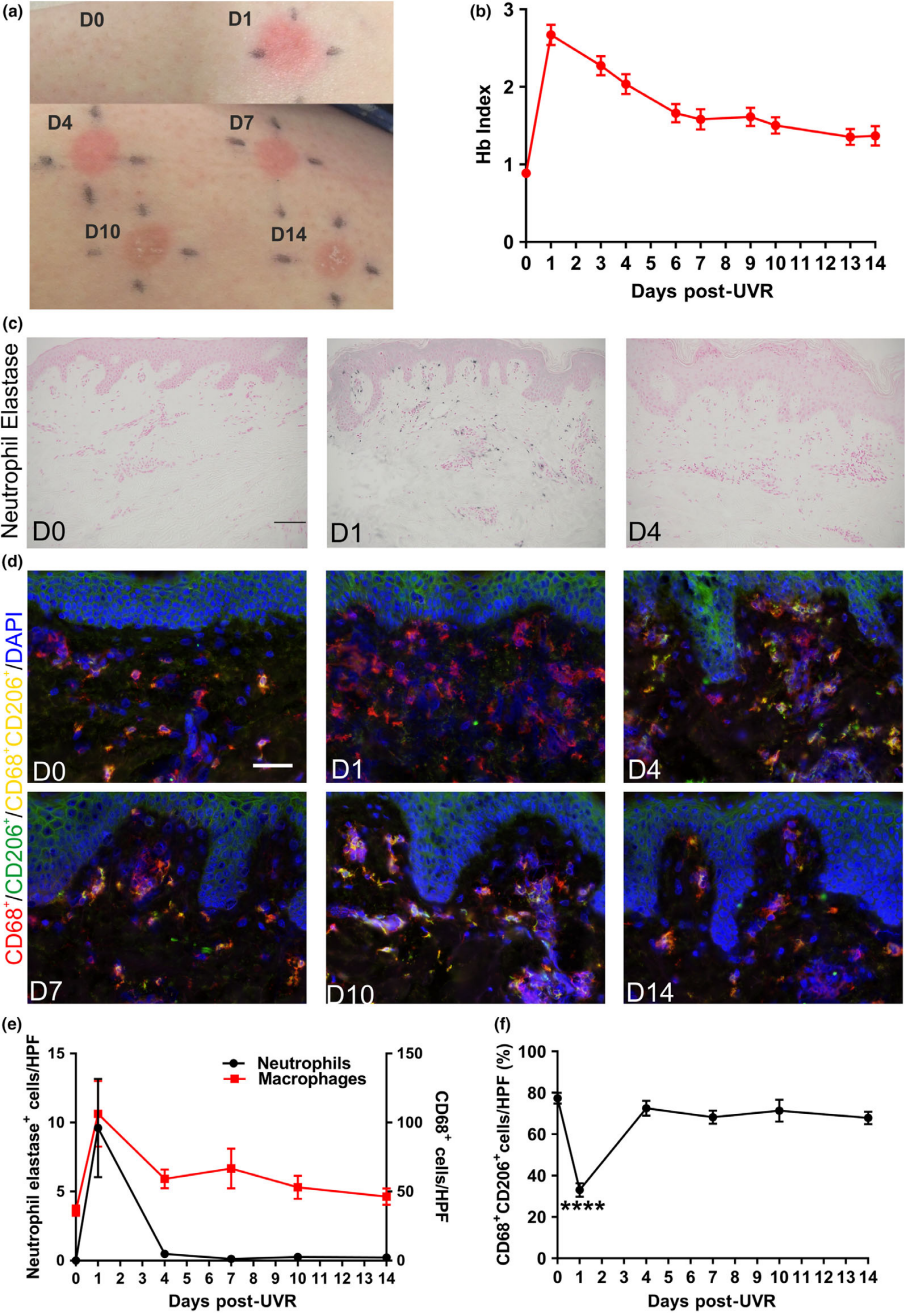
Classical cellular resolution (neutrophil clearance and macrophage polarisation) occurs at 4 days post‐UVR in human skin *in vivo.* **(a)** Macroscopic images of the clinical sunburn response in human skin up to 14 days post‐UVR exposure. **(b)** Spectroscopic quantification of Hb Index and resolution of erythema (*N* = 12 or 13 healthy volunteers for each time point). **(c)** Representative images of neutrophil elastase immunohistochemistry post‐UVR exposure. **(d)** Representative immunofluorescent images of M2 macrophage phenotyping (CD68^+^CD206^+^) post‐UVR exposure. **(e)** Quantification of neutrophil elastase and CD68 immunohistochemistry (*N* = 12 or 13 healthy volunteers for each time point). **(f)** Quantification of the percentage of M2 macrophages post‐UVR (*N* = 8 healthy volunteers for each time point). Data are mean ± SEM. Experiments were performed once. Scale bars = 50 µm. ****P < 0.0001.

To determine when resolution of the clinical sunburn response occurs, we analysed skin erythema following a single pro‐inflammatory dose of UVR [3 × minimal erythema dose (MED)]. The erythemal response to UVR, measured spectrophotometrically as Hb Index, was maximal at day 1 post‐challenge and decreased thereafter, returning to baseline levels by day 14, accompanied by visible tanning (Figure 1a and b).

Next, using immunohistochemistry (IHC) and immunofluorescence microscopy, we investigated whether classical cellular events of resolution applied to the UVR‐induced inflammatory response in humans, that is whereby infiltrating neutrophils are cleared and MФs progress to a M2‐like phenotype.^14^ At baseline, no neutrophils were detected, while total dermal neutrophil levels peaked at day 1 post‐UVR and returned to baseline levels at day 4 post‐UVR (Figure 1c and e). Using the pan‐Mφ marker, CD68, we detected dermal Mφs at baseline. After UVR provocation, Mφ numbers peaked at day 1 and returned to baseline levels at day 14 post‐UVR (Figure 1e, Supplementary figure 1).

Neutrophil numbers decreased to baseline after the peak of Mφ infiltration (Figure 1e), suggesting efferocytosis had taken place. The hallmark of efferocytosis is the active switching of the Mφ inflammatory phenotype (M1) to a more anti‐inflammatory (M2) status.^14^, ^15^ Therefore, we next wanted to determine whether the clearance of neutrophils corresponded with a change in Mφ phenotype. To do this, we used the M2 marker CD206 and quantified their phenotype in the dermis, since the majority of the Mφ infiltrate resided in this skin compartment (Supplementary figure 1). At baseline, ~77% of Mφs had an M2‐like phenotype, and following UVR exposure, a shift in phenotype was observed with a reduction (~33%) in M2‐like cells, suggesting the Mφ population was M1‐dominant (Figure 1d and f). Thereafter, on day 4 post‐UVR, dermal Mφs reverted back towards a baseline phenotype with ~72% of CD68^+^ Mφs expressing CD206 (Figure 1d and f).

**Figure 2:**
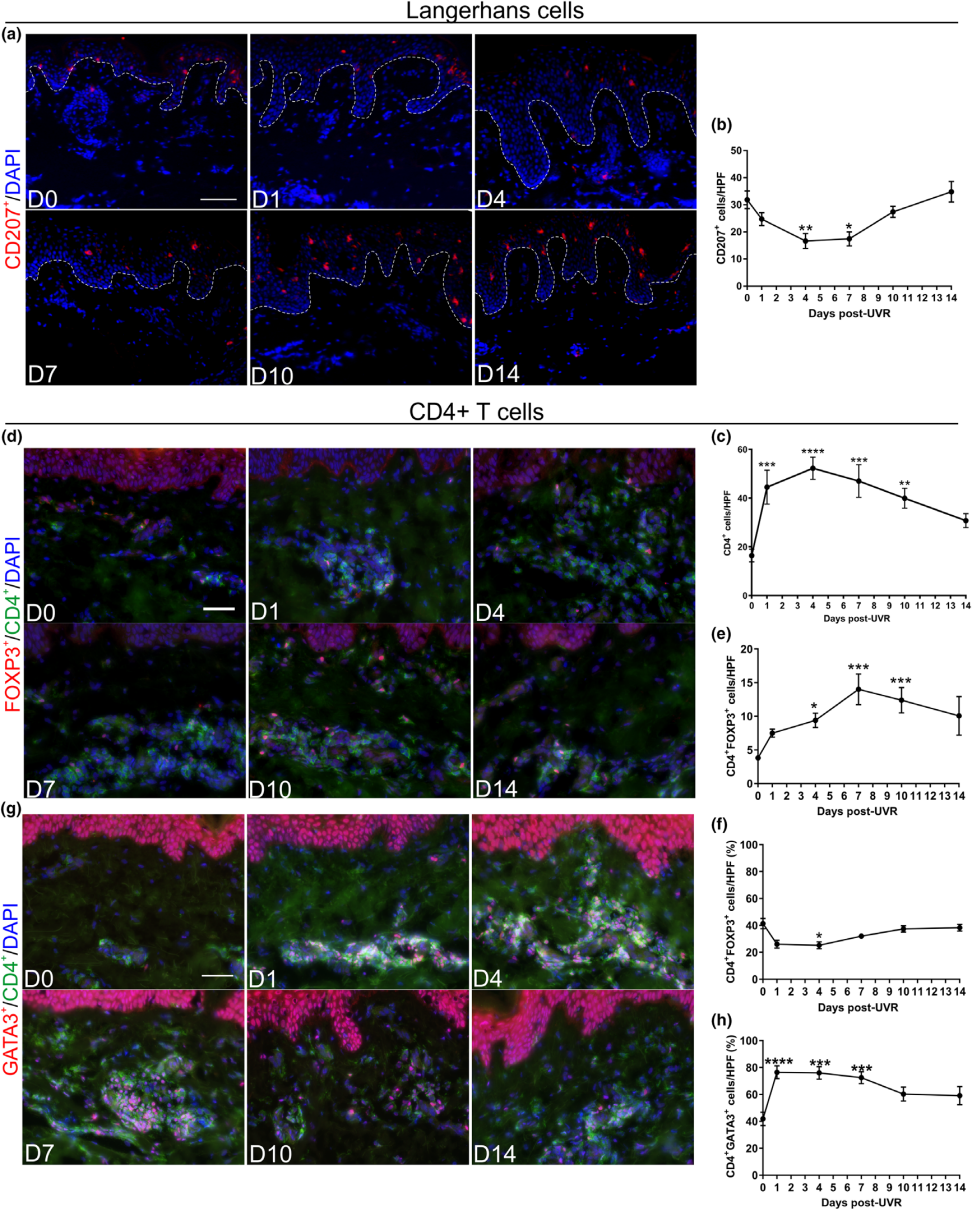
UVR‐induced inflammation recruits CD4^+^FOXP3^+^ T regulatory cells during the post‐resolution phase in human skin; however, CD4^+^GATA3^+^ T cells are the dominant phenotype*.* **(a)** Representative immunofluorescent images of CD207^+^ Langerhans cells post‐UVR. **(b)** Quantification of CD207^+^ post‐UVR exposure (*N* = 12 healthy volunteers for each time point). **(c)** Quantification of the total number of CD4^+^ cells on immunohistochemical staining (*N* = 12 or 13 healthy volunteers for each time point). **(d)** Representative immunofluorescent images of CD4^+^FOXP3^+^ cells post‐UVR **(e)** Quantification of the number of dual‐positive CD4^+^FOXP3^+^ cells post‐UVR (*N* = 8 healthy volunteers for each time point). **(f)** Percentage of CD4^+^FOXP3^+^ cells of total CD4^+^ cells post‐UVR (*N* = 8 healthy volunteers for each time point). **(g)** Representative immunofluorescent images of CD4^+^GATA3^+^ cells post‐UVR. **(h)** Percentage of CD4^+^GATA3^+^ cells of total CD4^+^ cells post‐UVR (*N* = 7 or 8 healthy volunteers for each time point). Data are mean ± SEM. Experiments were performed once. Scale bars = 50 µm. *P < 0.05, **P < 0.01, ***P < 0.001, ****P < 0.0001.

Thus, the temporal cellular changes in neutrophil and Mφ infiltration at the peak and resolution of UVR inflammation are similar to other forms of inflammation.^19^ However, the cellular events differed from the post‐resolution phase in mice and humans challenged with other inflammatory stimuli. Specifically, we did not observe a second infiltrate of Mφs at 14 days post‐UVR (Figure 1e, Supplementary figure 1). This indicates that other cells must participate in the post‐resolution phase of sunburn in human skin. To explore this, we first analysed mast cells through IHC and found no change throughout the time course (Supplementary figure 1). Conversely, total epidermal Langerhans cell (LC) numbers were significantly decreased (Figure 2a and b). As migrating LCs post‐UVR express co‐stimulatory molecules,^20^ this suggests that LCs are migrating to the lymph nodes to activate T cells to home to the site of UVR inflammation.

### CD4^+^GATA3^+^ T cells are the main CD4^+^ phenotype following UVR‐induced inflammation

Tregs are thought to facilitate Mφ efferocytosis^21^ and cutaneous photoimmunosuppression in mice.^11^, ^12^, ^22^ Therefore, we quantified dermal CD4^+^ T cells by IHC. CD4^+^ T cells were present in unexposed skin, and upon day 1 post‐UVR, a significant increase in their numbers was detected, followed by peak infiltration at day 4 and return to baseline levels by day 14 post‐UVR (Figure 2c, Supplementary figure 1). As CD4^+^ infiltrate is predominantly dermal (Supplementary figure 1), we quantified Tregs in the dermis using the marker FOXP3 with CD4. After UVR provocation, the number of CD4^+^FOXP3^+^ cells significantly increased by 4 days post‐UVR, the time point where we defined cellular resolution to occur (Figure 1), and peaked at 10 days post‐UVR (Figure 2d and e). While we detected a significant increase in the total number of Treg cells following UVR provocation, the percentage of CD4^+^FOXP3^+^ was reduced to ~ 25% from ~ 41% of CD4^+^ cells (Figure 2f). This contrasts with murine data where CD4^+^FOXP3^+^ T cells are the major subpopulation of CD4^+^ cells (~60%).^11^, ^12^ Therefore, in human skin, this infiltrate comprises other CD4^+^ effector T cells.

To investigate this, we quantified the Th2 marker, GATA3, in the dermis by immunofluorescence. This revealed a significant increase in the percentage of CD4^+^GATA3^+^ cells, demonstrating that CD4^+^GATA3^+^ cells are the dominant phenotype during the sunburn response in humans (Figure 2g and h). While GATA3 is a marker for Th2 cells, GATA3 can regulate anti‐inflammatory cytokines independently of Th2 cytokines.^23^ Since IL‐4 is expressed by neutrophils^24^ and not by CD3^+^ T cells^25^ post‐UVR in human skin, this suggests the CD4^+^GATA3^+^ cells we identified here mediate an immunosuppressive effect rather than a Th2 response. Therefore, both CD4^+^GATA3^+^ and CD4^+^FOXP3^+^ are likely to provide an immunosuppressive environment in human skin.

### UVR‐induced inflammation recruits and maintains CD8^+^GATA3^+^ T cells

**Figure 3:**
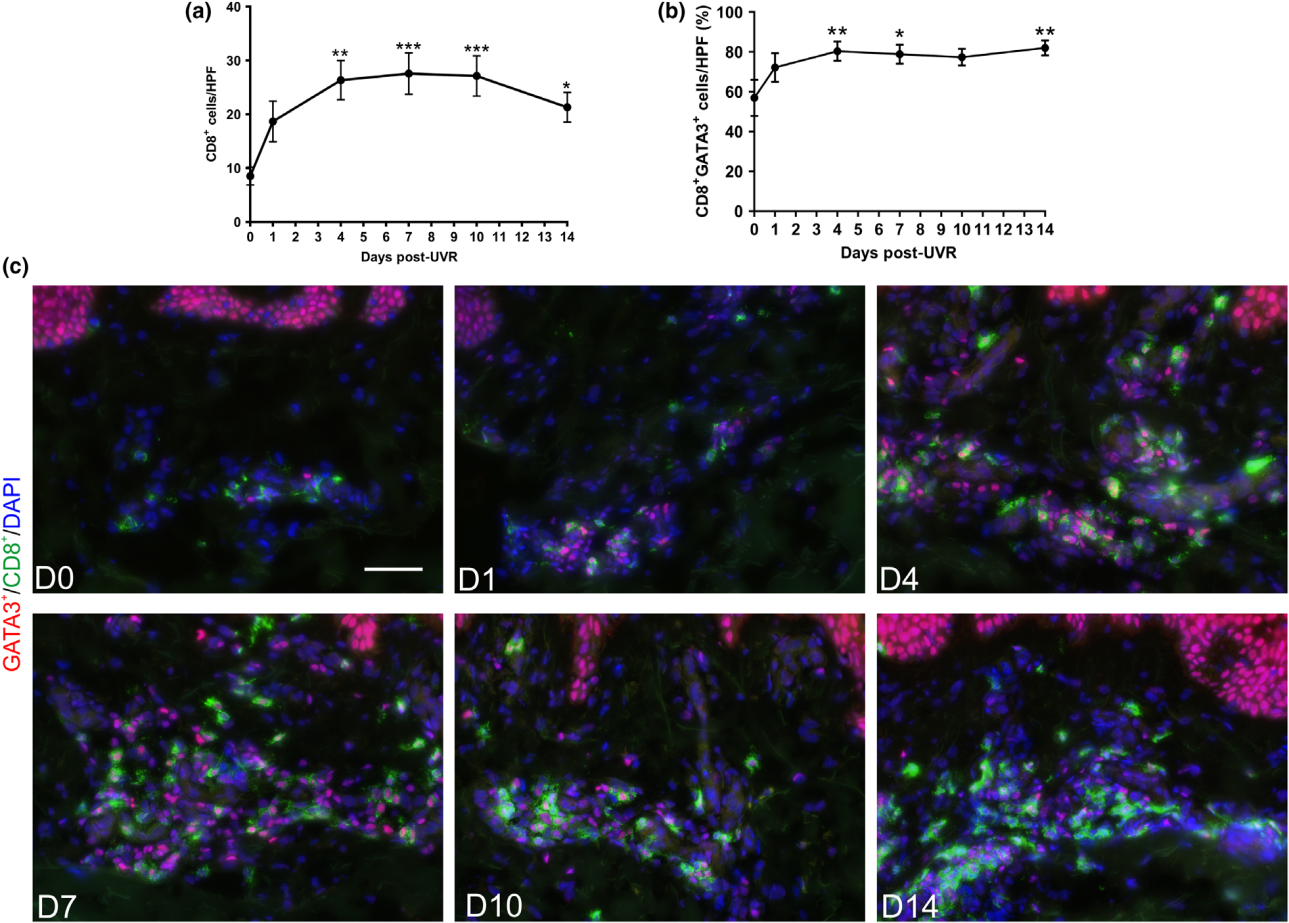
UVR‐induced inflammation recruits and maintains CD8^+^GATA3^+^ T cells 14 days post‐UVR*.* **(a)** Quantification of the total number of CD8^+^ cells on immunohistochemical staining (*N* = 12 or 13 healthy volunteers for each time point). **(b)** Percentage of CD8^+^GATA3^+^ cells of total CD8^+^ cells by immunofluorescence analysis (*N* = 8 healthy volunteers for each time point). **(c)** Representative immunofluorescent images of CD8^+^GATA3^+^ cells post‐UVR. Data are mean ± SEM. Experiments were performed once. Scale bars = 50 µm. *P < 0.05, **P < 0.01, ***P < 0.001.

The epidermis constitutively expressed GATA3, as anticipated since GATA3 is expressed by keratinocytes. Notably, we detected CD4^−^GATA3^+^ cells in the dermis; we hypothesised that these cells are the CD8 T‐cell subpopulation Tc2. To determine whether CD8 T cells have any role in the sunburn response, we analysed total dermal CD8^+^ cells by IHC (Figure 3a, Supplementary figure 1). This revealed CD8^+^ cells peaked 7 days post‐UVR and remained significantly elevated 14 days post‐UVR (Figure 3a, Supplementary figure 1). As CD8^+^ cells are almost exclusively restricted to the dermis, we quantified their GATA3 expression in the dermal compartment. At baseline, ~56% of CD8^+^ cells expressed GATA3 and these peaked 4 days post‐UVR (~80%) and remained significantly elevated at 14 days post‐UVR (~82%) (Figure 3b and c, Supplementary figure 2). This demonstrates that CD8^+^GATA3^+^ T cells are recruited and maintained following UVR exposure in human skin. Dysfunctional CD8^+^GATA3^+^ T cells are reported to occur upon chronic inflammation, losing their cytotoxic function and gaining an immunosuppressive profile,^26^ which potentially is the case here.

### UVR induces the synthesis of lipid mediators beyond cellular resolution in human skin *in vivo*

Lipid mediators tightly regulate the induction of inflammation through coordinating immune cell functions or immune profile,^15^ and facilitate resolution to prevent chronic inflammation.^10^, ^27^ However, it is unknown whether lipid mediators participate in the resolution or post‐resolution phases of the UVR response in humans. To investigate this, we analysed their temporal expression in suction blister fluid by LC‐MS/MS to 14 days post‐UVR.

**Figure 4:**
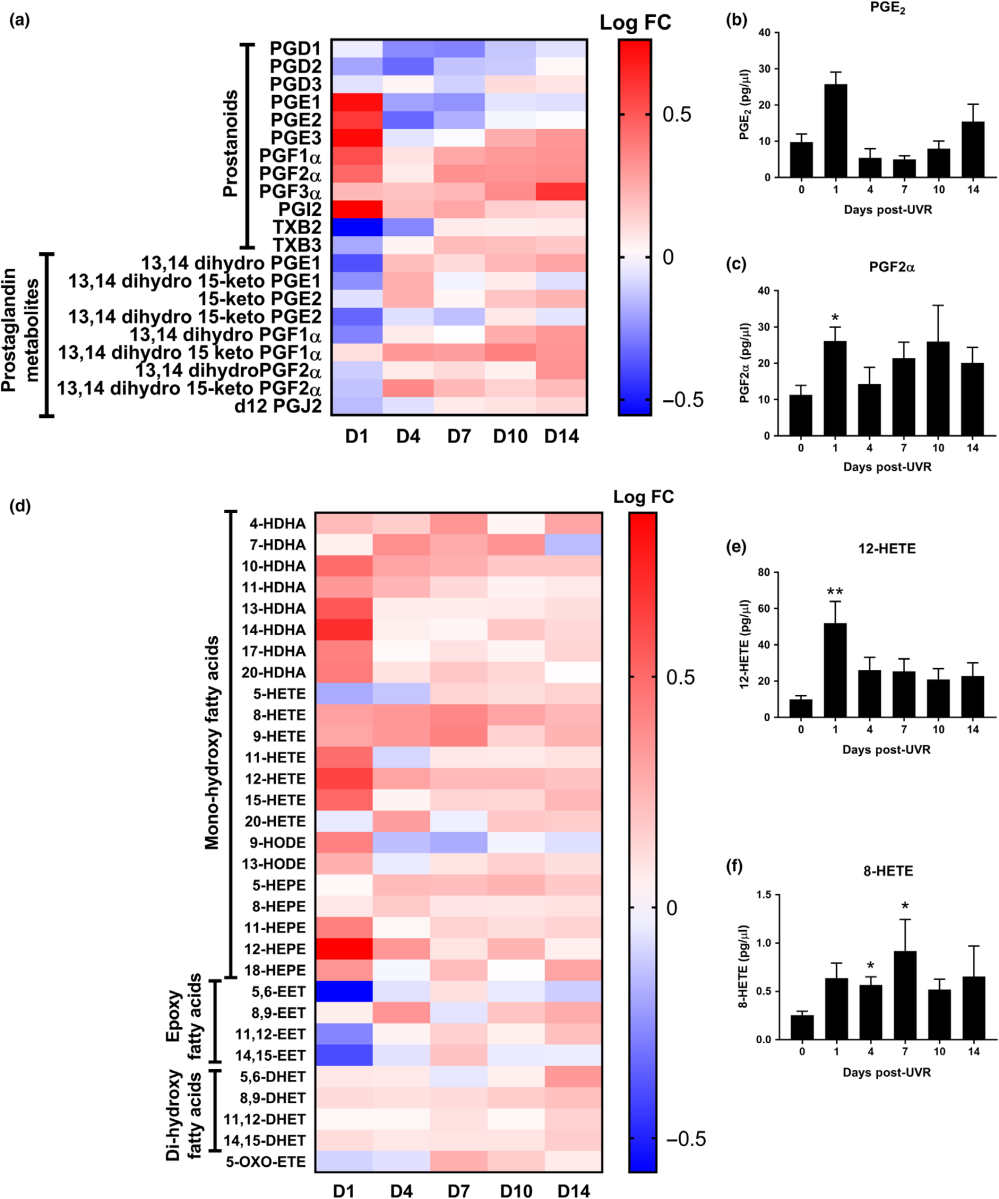
Prostanoid and hydroxy fatty acid synthesis continue up to 14 days post‐UVR in human skin *in vivo.* **(a)** Heat map of prostanoids and their breakdown products in human skin suction blister fluid following UVR exposure. **(b, c)** Selected prostanoids that demonstrate an increase at the peak of inflammation (PGE_2_) and during the post‐resolution phase of the sunburn response (PGF2_α_). **(d)** Heat map of hydroxy fatty acids in human skin suction blister fluid following UVR exposure. **(e, f)** Selected hydroxy fatty acids that demonstrate an increase at the peak of inflammation (12‐HETE) and during the post‐resolution phase (8‐HETE) (*N* = 10 or 12 healthy volunteers for each time point). Data are mean ± SEM. Experiments were performed once. *P < 0.05, **P < 0.01.

The production of many prostaglandins (Figure 4a and b) and mono‐hydroxy fatty acids (Figure 4d and e) increased 1 day post‐UVR, in line with the peak of the inflammatory sunburn response. By day 4 post‐UVR, pro‐inflammatory prostaglandin (PGE_1_‐PGE_3_) levels had normalised (Figure 4a, b, d and e), supporting our observation that cellular resolution occurs at 4 days post‐UVR. We were unable to detect the specialised pro‐resolving mediators known as resolvins, although we did identify their precursors 17‐HDHA and 18‐HEPE (Figure 4d).

Following resolution of UVR inflammation, certain prostaglandins continued to be synthesised, most obviously the PGF series (Figure 4a and c), indicating an active role in post‐resolution processes. In addition, prostaglandin metabolites increased 4 days post‐UVR. This may reflect increased synthesis of their precursors but some species such as d12‐PGJ_2_ exhibit anti‐inflammatory activities in their own right.^28^ We also detected prolonged synthesis of hydroxy fatty acids (Figure 4d), in particular the chemoattractant 8‐HETE at days 4 and 7 post‐UVR (Figure 4f), which may be associated with the T‐cell infiltrate at this time. Therefore, the skin lipid microenvironment does not simply revert to its tissue setting prior to UVR provocation, indicating that adaptive homeostasis^8^, ^10^ applies to UVR exposure in humans.

### Concluding remarks

Collectively, we demonstrate for the first time in humans *in vivo* that a single exposure to UVR has long‐lasting effects on the recruitment and maintenance of a dermal immune infiltrate and the skin microenvironment. These findings offer explanation for the clinical observation of immunosuppression provoked by UVR,^3^ with insights into the events that lead to resolution of UVR injury, and the differences in post‐resolution biology occurring in human skin.

Interestingly, our results demonstrate discrepancies between the human and murine adaptive immune response to UVR. In mice, Tregs are the dominant CD4^+^ T cells post‐UVR; however, here, they did not constitute the major CD4^+^ population. Instead, we identified that a substantial proportion of CD4^+^ T cells expressed the transcription factor GATA3. Therefore, our results caution against extrapolation of murine mechanistic studies of the cutaneous UVR response. Furthermore, we detected a retention of CD8^+^GATA3^+^ T cells following UVR inflammation, which has been previously overlooked with regard to photoimmunology.^22^ As this CD8^+^ subset has immunosuppressive activities through GATA3 increasing IL‐10 production,^26^ CD8^+^GATA3^+^ T cells may have a physiological role for post‐resolving events following acute UVR‐induced inflammation. Therefore, our work introduces GATA3 as a potential mediator of the adaptive immune response to UVR in human skin. While we have identified these cells for the first time in human skin following UVR inflammation, functional analyses are awaited and further immune cells may be contained within the infiltrate.

Finally, the concept that prolonged immunosuppression after an initiating inflammatory stimulus is beneficial in preventing ‘maladapted homeostasis’^8^ may extend to UVR injury in humans, as we detected substantial immune activity 14 days post‐UVR. Specifically, prostaglandin and hydroxy fatty acid synthesis were elevated and CD8^+^GATA3^+^ T cells were maintained after resolving cellular (neutrophilic) events. These cells and mediators may be necessary to conserve an immunosuppressive environment and prevent chronic inflammation or autoimmunity, as there is considerable release of potential self‐antigens following UVR.^29^ Targeting the activity of these immune cells (e.g. CD4^+^/CD8^+^GATA3^+^ cells to increase IL‐10, or M2 MФs to augment efferocytosis) or manipulating the synthesis of lipid mediators may enhance inflammation resolution and optimise the post‐resolution microenvironment following UVR exposure.

## Methods

### Subjects

Ethical approval was granted by the Greater Manchester North NHS research ethics committee (ref: 11/NW/0567). The study took place at the Photobiology Unit, Salford Royal NHS Trust, Greater Manchester, UK, and 13 healthy volunteers were recruited from the local area. Potential volunteers were included if they were aged between 18 and 60 years, white Caucasian, of skin phototypes I‐III according to the Fitzpatrick skin phototyping scale. Volunteer suitability was also determined through assessment of their sunburn threshold, that is minimal erythemal dose (MED; see below). Exclusion criteria were as follows: pregnant/breastfeeding, had used a sunbed or sunbathed in the 3 months prior to the study, had pre‐existing skin conditions, history of skin cancer, or taking anti‐inflammatory medication or supplements. All volunteers provided written informed consent in accordance with the Declaration of Helsinki principles.

### Ultraviolet irradiation challenge

Each volunteer’s MED was determined by application of 10 increasing doses (8, 10, 13, 17, 21, 32, 42, 51, 63 and 80 mJ cm^−2^) of UVR to the photoprotected upper buttock using a Waldmann UV 236B unit housing two Waldman fluorescent broadband UVB lamps (280–400 nm; peak 313 nm; Herbert Waldmann GmbH & Co. KG, Germany). The dose producing a just visible erythema at 24 h post‐UVR exposure was the volunteer’s MED. Along with the skin phototype, the MED result was used to determine suitability for inclusion in the study. Only volunteers with an MED of 42 mJ cm^−2^ or under were included in order to limit the variability in UVR‐induced inflammatory responses and select a group with greater sensitivity to UVR exposure. Volunteers who met these criteria were subsequently given a single individualised pro‐inflammatory dose of UVR (3 × MED) in duplicate to different sites of the photoprotected upper buttock skin on five different days (at the same time of day ± 2 h). This enabled erythema measurement and tissue sampling (punch biopsy and suction blister) of the 14‐day UVR time course.

### UVR‐induced erythema measurements

Volunteers were acclimatised for 15 min prior to taking Hb Index readings from unexposed and UVR‐exposed skin sites using a spectrophotometer (CM600d; Konica Minolta Sensing Europe, Warrington, UK). The Hb Index was measured at each of the skin sites exposed to a 3× MED UVR challenge, at each visit over a 14‐day period. The % resolution of the erythemal response over the 14‐day time course was calculated relative to the time point (day; *D*) showing the maximal Hb Index: ((Hb Index *D*_(max)_ – Hb Index *D*_(x)_/ Hb Index *D*_(max)_) × 100. In seven volunteers, Hb Index measurements were also taken at each of the 10 UVR‐exposed sites of the MED test at each visit over a 14‐day period. As the spectrophotometer simultaneously measures skin lightness (L*) using the CIE colour system, L* values of unexposed skin were also recorded for each individual at the beginning of the study.

### Skin sampling

Suction blisters were raised under negative pressure (250 mm Hg) on five UVR‐exposed sites and one unexposed skin site of the upper buttock using commercially available hand‐held vacuum pumps (Mityvac, St. Louis, MO, USA) and Perspex suction cups with a 1 cm diameter aperture (Medical Physics department, Salford Royal Foundation Trust, Greater Manchester, UK). Blister fluid was aspirated, snap‐frozen and stored at −80°C until analysis. Skin punch biopsies (5 mm) were taken from five UVR‐exposed sites and from one unexposed site under local anaesthetic (2% lidocaine; Antigen Pharmaceuticals Ltd, Tipperary, Ireland) using Militex biopsy punches (Militex Inc., York, PA, USA). Skin biopsies were bisected: half snap‐frozen in OCT medium and half fixed in 4% buffered formalin (Cell Path Ltd, Powys, Wales, UK) before processing in paraffin wax.

### Immunohistochemistry

Formalin‐fixed paraffin‐embedded skin samples were sectioned at 5 μm thickness. Sections were rehydrated, and endogenous peroxidase activity was blocked using 0.3% hydrogen peroxide in 1× phosphate‐buffered saline (PBS). Sections were incubated with monoclonal antibodies following heat‐induced antigen retrieval in citrate buffer (pH 6.0) (anti‐CD8, clone C8/144B; Dako, Stockport, UK); anti‐mast cell tryptase (clone AA1, Abcam, Cambridge, UK) or Tris‐EDTA buffer (pH 9) (anti‐CD4, clone 4B12, Dako). No antigen retrieval was required for neutrophil elastase staining (clone NP57, Dako). Frozen skin samples were sectioned at 7 μm thickness and used for CD68 (clone EBM11, Dako) staining. Primary antibody binding was visualised using Vector ImmPress (Vector Laboratories, Peterborough, UK) and Dako Envision (Dako) peroxidase kits according to the manufacturer’s instructions, and sections were counterstained using either nuclear fast red (Vector Laboratories) or Mayer’s haematoxylin (Sigma‐Aldrich Company Ltd, Dorset, UK). Images were acquired using a 20×/0.80 Plan Apo objective using the 3D Histech Pannoramic 250 Flash II slide scanner. Leucocytes were quantified by counting the number of positively stained cells in three high‐power fields (hpf) for neutrophils, CD8^+^ T cells, CD68^+^ macrophage and mast cells (×200) and in four hpf for CD4^+^ T cells (×400). Counts were performed in three skin sections per biopsy and averages calculated. Each IHC marker was quantified by one person, and this was performed in a blinded fashion.

#### Dual immunofluorescence

#### M2 MФs

Frozen skin blocks were sectioned at 7 µm and fixed in acetone for 10 min at −20°C, and then washed with Tris‐buffered saline [TBS (all wash steps used TBS)]. Sections were washed and incubated with 10% normal goat serum (NGS) for 30 min. Next, sections were incubated with anti‐CD68 (1:50 in 2% NGS; CD68, clone EBM11, Dako) and anti‐CD206 (1:100 in 2% NHS; anti‐mannose receptor, ab64693, Abcam) primary antibodies overnight (4 °C). The following day, sections were washed and incubated with goat anti‐mouse 594 (1:400 in 2% NGS; Alexa Fluor‐594, REF A11032, Thermo Fisher Scientific) and goat anti‐rabbit 488 (1:200 in 2% NGS; Alexa Fluor‐488, REFA11008, Thermo Fisher Scientific, Loughborough, UK.) secondary antibodies. Sections were washed, and nuclei were counterstained using DAPI (1 μg mL^−1^ in PBS) for 1 min. For negative controls, primary antibody raised against CD68 and CD206 was omitted. Images were acquired using an Olympus BX53 upright microscope (Olympus, Tokyo, Japan). MФs were quantified by counting the number of positively stained cells in three hpfs. Counts were performed in three skin sections per biopsy and averages calculated.

#### T‐cell phenotypes

Frozen skin blocks were sectioned at 7 µm and fixed in 4% PFA for 20 min at 4°C, and then washed with TBS (all wash steps used TBS). Sections were washed and incubated with 2% normal horse serum (NHS; S‐2012, Vector Laboratories) for 20 min. Next, sections were incubated with anti‐CD4 (1:50 in NHS; MA5‐12259, Thermo Fisher) or anti‐CD8 (1: 100 in NHS; MA5‐13473, Thermo Fisher) and in conjunction with anti‐FOXP3 (1:200 in NHS; ab4728, Abcam) or anti‐GATA3 (1:50 in NHS; ab199428, Abcam) primary antibodies overnight (4 °C). The following day, sections were washed and incubated with anti‐mouse excel DyLight 488 (DK‐2488; Vector Laboratories) and anti‐rabbit DyLight 594 (DI‐1794; Vector Laboratories) secondary antibodies. Sections were washed, and nuclei were counterstained using DAPI (1 μg mL^−1^ in PBS) for 1 min. For negative controls, primary antibody raised against CD4, CD8, FOXP3 and GATA3 was omitted. Images were acquired using an Olympus BX53 upright microscope (Olympus, Tokyo, Japan). Leucocytes were quantified by counting the number of positively stained cells in three hpfs. Counts were performed in three skin sections per biopsy and averages calculated.

#### Lipidomics

Suction blister fluid was analysed by mass spectrometry for prostanoid and hydroxy fatty acid species as previously described.^30^

### Statistics

Data were assessed for normality using D’Agostino–Pearson omnibus normality test and equal variance using F‐test. For analysis of parametric data, a one‐way ANOVA was performed with Dunnett test to correct for multiple comparisons, while the Kruskal–Wallis test was selected for nonparametric data with Dunn’s test to correct for multiple comparisons. If repeated measures were available, paired one‐way ANOVA was selected with either Holm–Sidak’s multiple comparisons test for normally distributed data or Dunn’s multiple comparisons test for nonparametric data. *P*‐values < 0.05 were considered significant. All statistical analyses were carried out in GraphPad Prism version 7 (GraphPad Software, La Jolla, CA, U.S.A.).

## Conflict of interest

The authors declare no conflict of interest.

## References

[1] Lucas RM , Yazar S , Young AR *et al* Human health in relation to exposure to solar ultraviolet radiation under changing stratospheric ozone and climate. Photochem Photobiol Sci 2019; 18: 641–680. 30810559 10.1039/c8pp90060d

[2] Guy GP , Machlin SR , Ekwueme DU *et al* Prevalence and costs of skin cancer treatment in the U.S., 2002–2006 and 2007–2011. Am J Prev Med 2015; 48: 183–187. 25442229 10.1016/j.amepre.2014.08.036 PMC4603424

[3] Kelly DA , Young AR , McGregor JM *et al* Sensitivity to sunburn is associated with susceptibility to ultraviolet radiation‐induced suppression of cutaneous cell‐mediated immunity. J Exp Med 2000; 191: 561–566. 10662801 10.1084/jem.191.3.561 PMC2195812

[4] Hart PH , Norval M , Byrne SN *et al* Exposure to ultraviolet radiation in the modulation of human diseases. Annu Rev Pathol Mech Dis 2019; 14: 55–81. 10.1146/annurev-pathmechdis-012418-012809 30125148

[5] Bais AF , Lucas RM , Bornman JF *et al* Environmental effects of ozone depletion, UV radiation and interactions with climate change: UNEP Environmental Effects Assessment Panel, update 2017. Photochem Photobiol Sci 2018; 17: 127–179. 29404558 10.1039/c7pp90043k PMC6155474

[6] Farr PM , Diffey BL . The erythemal response of human skin to ultraviolet radiation. Br J Dermatol 1985; 113: 65–76. 4015971 10.1111/j.1365-2133.1985.tb02045.x

[7] Bernard JJ , Gallo RL , Krutmann J . Photoimmunology: how ultraviolet radiation affects the immune system. Nat Rev Immunol 2019; 19: 688–701. 31213673 10.1038/s41577-019-0185-9

[8] Feehan KT , Gilroy DW . Is resolution the end of inflammation? Trends Mol Med 2019; 25: 198–214. 30795972 10.1016/j.molmed.2019.01.006

[9] Newson J , Stables M , Karra E *et al* Resolution of acute inflammation bridges the gap between innate and adaptive immunity. Blood 2014; 124: 1748–1764. 25006125 10.1182/blood-2014-03-562710 PMC4383794

[10] Newson J , Motwani MP , Kendall AC *et al* Inflammatory resolution triggers a prolonged phase of immune suppression through COX‐1/mPGES‐1‐derived prostaglandin E_2_ . Cell Rep 2017; 20: 3162–3175. 28954232 10.1016/j.celrep.2017.08.098 PMC5639146

[11] Yamazaki S , Odanaka M , Nishioka A *et al* Ultraviolet B‐induced maturation of CD11b‐type langerin − dendritic cells controls the expansion of Foxp3^+^ regulatory T cells in the skin. J Immunol 2018; 200: 119–129. 29158419 10.4049/jimmunol.1701056

[12] Yamazaki S , Nishioka A , Kasuya S *et al* Homeostasis of thymus‐derived Foxp3^+^ regulatory T cells is controlled by ultraviolet B exposure in the skin. J Immunol 2014; 193: 5488–5497. 25348622 10.4049/jimmunol.1400985

[13] Hesterberg RS , Amorrortu RP , Zhao Y *et al* T Regulatory cell subpopulations associated with recent ultraviolet radiation exposure in a skin cancer screening cohort. J Immunol 2018; 201: 3269–3281. 30389774 10.4049/jimmunol.1800940 PMC6941348

[14] Fullerton JN , Gilroy DW . Resolution of inflammation: a new therapeutic frontier. Nat Rev Drug Discov 2016; 15: 551. 27020098 10.1038/nrd.2016.39

[15] Buckley CD , Gilroy DW , Serhan CN . Proresolving lipid mediators and mechanisms in the resolution of acute inflammation. Immunity 2014; 40: 315–327. 24656045 10.1016/j.immuni.2014.02.009 PMC4004957

[16] Serhan CN , Levy BD . Resolvins in inflammation: emergence of the pro‐resolving superfamily of mediators. J Clin Invest 2018; 128: 2657–2669. 29757195 10.1172/JCI97943 PMC6025982

[17] Rhodes LE , Gledhill K , Masoodi M *et al* The sunburn response in human skin is characterized by sequential eicosanoid profiles that may mediate its early and late phases. FASEB J 2009; 23: 3947–3956. 19584301 10.1096/fj.09-136077 PMC2791058

[18] Rhodes LE , Belgi G , Parslew R *et al* Ultraviolet‐B‐induced erythema is mediated by nitric oxide and prostaglandin E2 in combination. J Invest Dermatol 2001; 117: 880–885. 11676827 10.1046/j.0022-202x.2001.01514.x

[19] Motwani MP , Flint JD , De Maeyer RPH *et al* Novel translational model of resolving inflammation triggered by UV‐killed E. coli. J Pathol Clin Res 2016; 2: 154–165. 27499924 10.1002/cjp2.43 PMC4958736

[20] Achachi A , Vocanson M , Bastien P *et al* UV radiation induces the epidermal recruitment of dendritic cells that compensate for the depletion of langerhans cells in human skin. J Invest Dermatol 2015; 135: 2058–2067. 25806853 10.1038/jid.2015.118

[21] Proto JD , Doran AC , Gusarova G *et al* Regulatory T cells promote macrophage efferocytosis during inflammation resolution. Immunity 2018; 49: 666–677.e6. 30291029 10.1016/j.immuni.2018.07.015 PMC6192849

[22] Schwarz T , Beissert S . Milestones in Photoimmunology. J Invest Dermatol 2013; 133: E7–E10. 10.1038/skinbio.2013.177 23820723

[23] Shoemaker J , Saraiva M , O’Garra A . GATA‐3 directly remodels the IL‐10 locus independently of IL‐4 in CD4^+^ T cells. J Immunol 2006; 176: 3470–3479. 16517715 10.4049/jimmunol.176.6.3470

[24] Teunissen MBM , Piskin G , di Nuzzo S *et al* Ultraviolet B radiation induces a transient appearance of IL‐4^+^ neutrophils, which support the development of Th2 responses. J Immunol 2002; 168: 3732–3739. 11937523 10.4049/jimmunol.168.8.3732

[25] Di Nuzzo S , Sylva‐Steenland RMR , Koomen CW *et al* UVB irradiation of normal human skin favors the development of type‐2 T‐cells in vivo and in primary dermal cell cultures. Photochem Photobiol 2002; 76: 301–309. 12403451 10.1562/0031-8655(2002)076<0301:uionhs>2.0.co;2

[26] Singer M , Wang C , Cong L *et al* A distinct gene module for dysfunction uncoupled from activation in tumor‐infiltrating T cells. Cell 2016; 166: 1500–1511. 27610572 10.1016/j.cell.2016.08.052 PMC5019125

[27] Motwani MP , Newson J , Kwong S *et al* Prolonged immune alteration following resolution of acute inflammation in humans. PLoS One 2017; 12: e0186964. 29073216 10.1371/journal.pone.0186964 PMC5658111

[28] Straus DS , Glass CK . Cyclopentenone prostaglandins: new insights on biological activities and cellular targets. Med Res Rev 2001; 21: 185–210. 11301410 10.1002/med.1006

[29] Bernard JJ , Cowing‐Zitron C , Nakatsuji T *et al* Ultraviolet radiation damages self noncoding RNA and is detected by TLR3. Nat Med 2012; 18: 1286–1290. 22772463 10.1038/nm.2861 PMC3812946

[30] Pilkington SM , Rhodes LE , Al‐Aasswad NMI *et al* Impact of EPA ingestion on COX‐ and LOX‐mediated eicosanoid synthesis in skin with and without a pro‐inflammatory UVR challenge ‐ report of a randomised controlled study in humans. Mol Nutr Food Res 2014; 58: 580–590. 24311515 10.1002/mnfr.201300405 PMC4377077

