# Supplementary material for: UV radiation recruits CD4+GATA3+ and CD8+GATA3+ T cells while altering the lipid microenvironment following inflammatory resolution in human skin in vivo
Source: Clin Transl Immunology. 2020 Apr 2;9(4):e01104. doi: 10.1002/cti2.1104 (PMC7114692; doi:10.1002/cti2.1104)
Supplement: Supplementary file 1 [file CTI2-9-e01104-s001.docx]

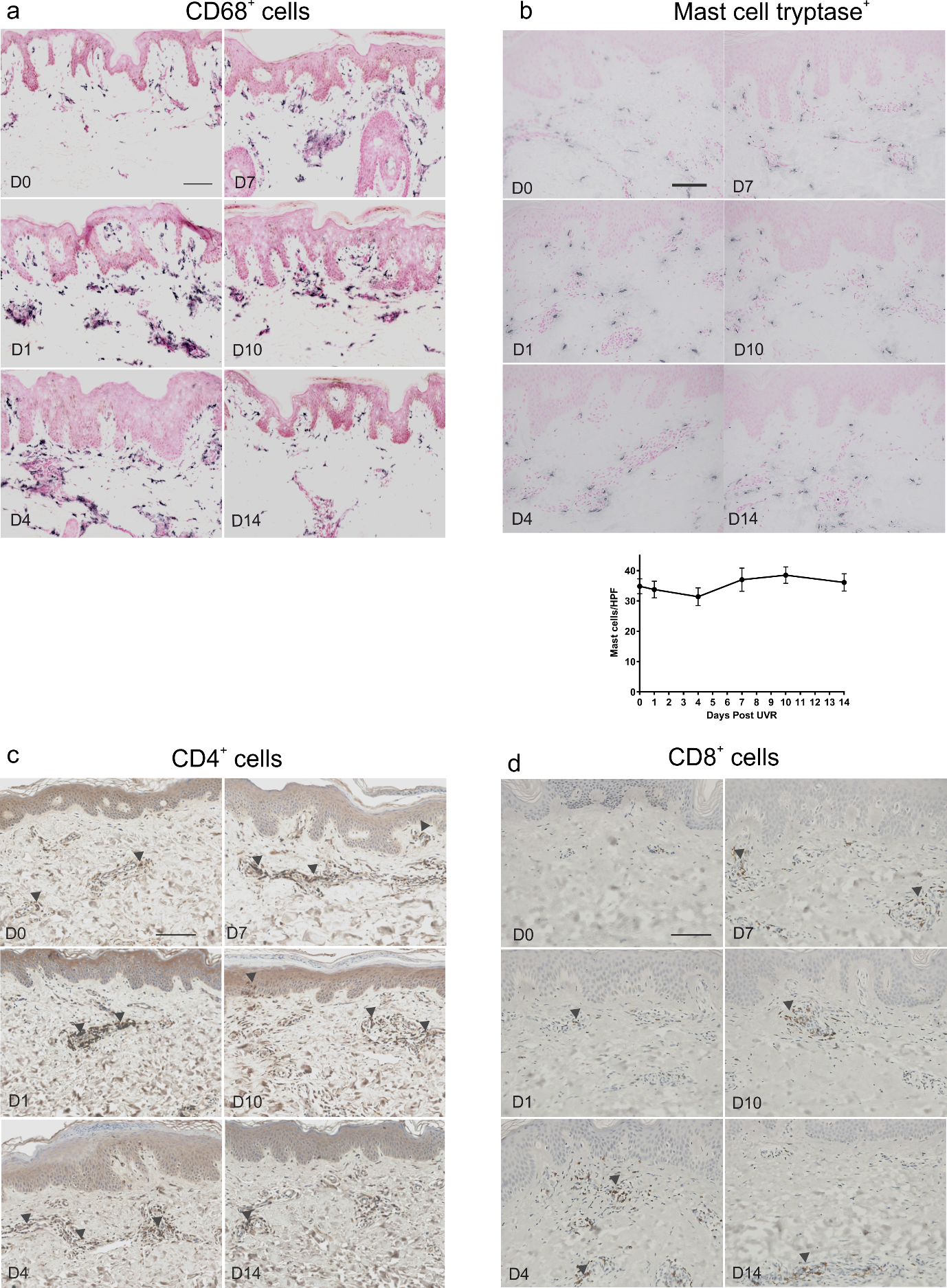


**Supplementary Figure 1**. Immunohistochemistry of immune cell markers following UVR-induced inflammation in healthy human skin *in vivo*. (a) Representative images of CD68^+^ cells. (b) Representative images and quantification of mast cell tryptase^+^ cells. (c) Representative images of CD4^+^ cells. (d) Representative images of CD8^+^ cells. *N* = 10 or 11 healthy volunteers for each time point. Data are mean ± SEM.
