# Supplementary material for: UV radiation recruits CD4+GATA3+ and CD8+GATA3+ T cells while altering the lipid microenvironment following inflammatory resolution in human skin in vivo
Source: Clin Transl Immunology. 2020 Apr 2;9(4):e01104. doi: 10.1002/cti2.1104 (PMC7114692; doi:10.1002/cti2.1104)
Supplement: Supplementary file 2 [file CTI2-9-e01104-s002.docx]

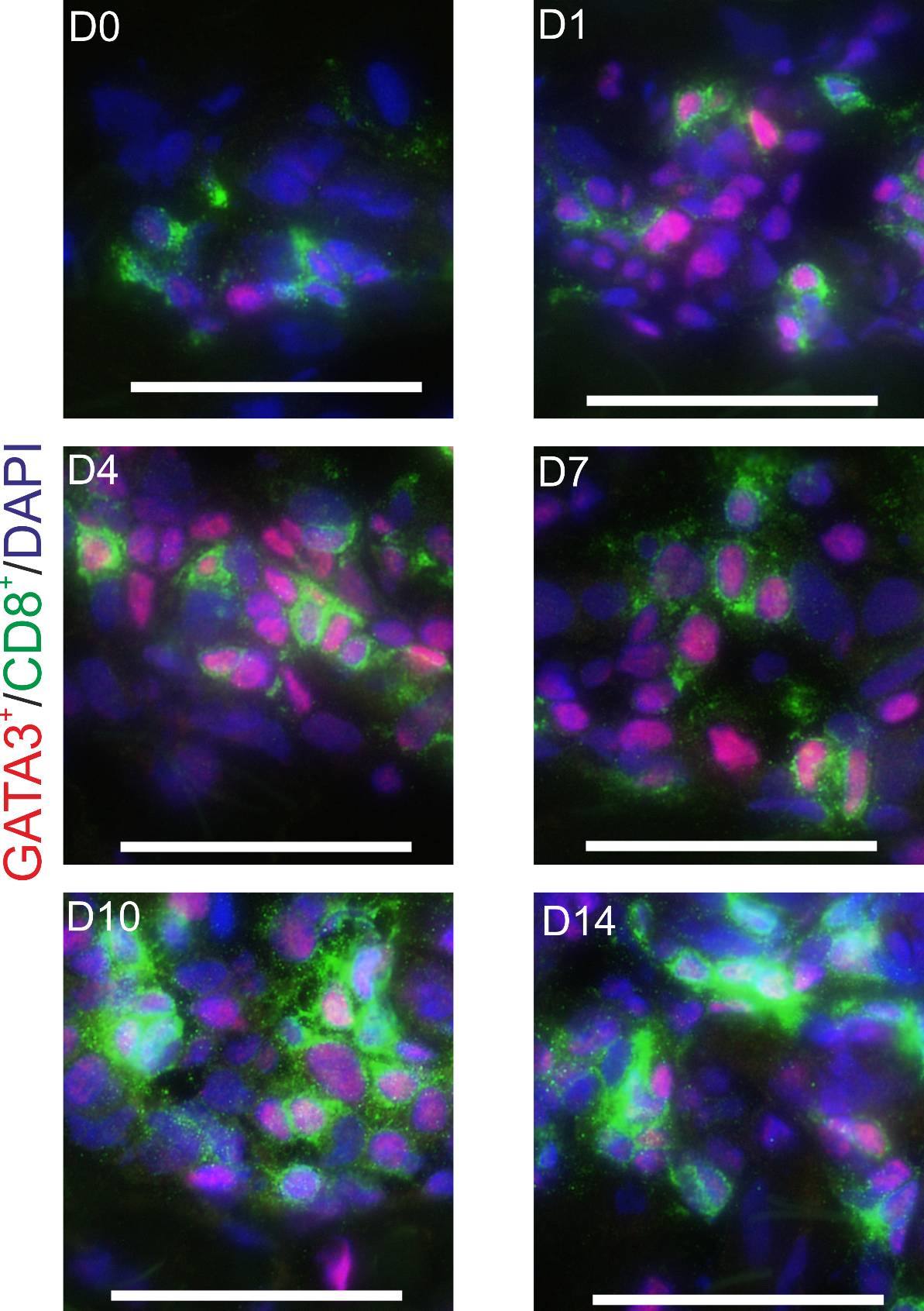


**Supplementary Figure 2**. High magnification of dual positive CD8^+^GATA3^+^ T-cells following UVR-induced inflammation in healthy human skin *in vivo*. Scale bars = 50 µm.
